# Isometric Double-Layer Staggered Chain Teeth Triboelectric Nanogenerator

**DOI:** 10.3390/mi13030421

**Published:** 2022-03-08

**Authors:** Shuai Ding, Hua Zhai, Yaomin Shao, Rui Lei

**Affiliations:** Anhui Province Key Lab of Aerospace Structural Parts Forming Technology and Equipment, Institute of Industry & Equipment Technology, Hefei University of Technology, Hefei 230009, China; 13201530378@163.com (S.D.); q1170538712@163.com (Y.S.); leirui2078@163.com (R.L.)

**Keywords:** triboelectric nanogenerator, double layer, staggered electrode, chain teeth

## Abstract

The sliding freestanding layer triboelectric nanogenerator (SF-TENG) is a sustainable power source that can convert mechanical energy from linear or rotating mechanical motion to electrical energy. This paper proposes a double-layer staggered chain teeth TENG. Comparing the staggered electrode TENG and the double-layer staggered electrode TENG, the output voltage difference is relatively small. The electrode of the TENG is designed to the shape of chain teeth, which proves that TENG can be combined with a zipper, and the best distance among chain teeth in the TENG is determined through experiments. Compared with traditional zippers, the double-layer staggered chain teeth TENG can generate electrical energy during the continuous pulling of the zipper. The double-layer staggered chain teeth TENG has good performance. When the external load is 20 MΩ, the maximum output power reaches 20.18 µW. After the rectification and transformation, the generated electricity can light up 30 LED lights or more, and can also supply power to electronic devices. Through the chain teeth array, the open circuit voltage and transfer charge generated by the zipper during the continuous pulling process are improved. The double-layer staggered chain teeth TENG has a good usage environment in life, and this work will provide valuable insights for the development of SF-TENG technology.

## 1. Introduction

With the rapid development of Internet of Things technology and smart electronic devices, the replacement time of electronic products is becoming shorter and shorter, and electronic devices are gradually developing towards miniaturization, mobility, and multi-function. Realizing the self-powered operation of smart devices is an ideal alternative, which can avoid problems, such as extra economic burden and environmental pollution caused by the regular replacement of power supplies for battery-driven electronic equipment [1,2]. Nowadays, the method of using the piezoelectric effect, electromagnetic induction and friction effect to collect micro-nano energy in the environment as a sustainable power source has received extensive attention. The electrostatic charge generated in the process of triboelectricity has been considered a negative effect for several years due to its easy attraction to dust, and because it causes electrical discharges and short circuits. It is generally considered that the electrostatic charge generated by friction is a kind of wasted electrical energy [3]. In 2012, Professor Zhonglin Wang invented triboelectric nanogenerator (TENG) technology based on triboelectric and electrostatic induction coupling [4,5,6,7,8], which can collect various forms of energy and convert it into electrical energy, such as wind energy [9,10,11,12,13], ocean energy [14,15,16], vibration energy [17,18,19], human movement energy [20,21,22]. Compared with the traditional electromagnetic induction power generation technology, TENG has the advantages of being light weight and having a high-power generation voltage, small size, flexibility and shape flexibility, and high compatibility [23,24,25,26], and has received extensive attention from researchers.

According to the working mode, TENG can be divided into four types: contact separation (CS-TENG) [27,28], horizontal sliding (S-TENG) [29,30], single electrode (SE-TENG) [31] and freestanding triboelectric layer (F-TENG) [32,33,34]. Vertical linear motion usually has CS-TENG and SE-TENG modes, while sliding linear motion has S-TENG, SE- TENG and F-TENG modes [35,36,37].

This paper proposes a double-layer staggered chain teeth TENG, and conducts experiments to explore the feasibility of the conjecture. The double-layer staggered chain teeth TENG is composed of multiple units, and each unit is composed of several chain teeth made of acrylic plates. All the chain teeth are plated with a copper film to serve as electrodes for TENG. The freestanding layer framework is made of acrylic material that is covered with a sponge, and a polyimide (Kapton) film is pasted on the sponge to serve as the freestanding layer. The main purpose of sticking the sponge on the surface is to increase the close contact between the Kapton and the electrode, so that the freestanding layer can be in close contact with the electrode, and a greater output effect can be obtained. The TENG unit follows the working principle of the independent mode. Compared with the double-layer staggered electrode TENG, the double-layer staggered chain teeth TENG has a slightly smaller output effect. According to the experiment, the best chain teeth distance of the double-layer staggered chain teeth TENG is obtained. The double-layer staggered chain teeth TENG is similar in shape to the zipper, which makes it possible to combine TENG with the zipper. When people encounter no electricity outdoors, they can pull the zipper on the bag or clothes to start TENG work to charge the internally connected capacitors and the charged capacitors can be used in some emergency situations.

## 2. Methods

### 2.1. Basic Theoretical Methods

**Figure 1:**
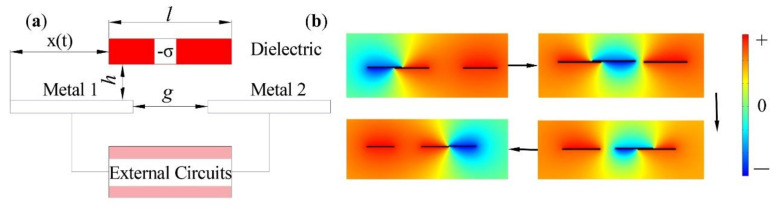
(**a**) Model of the freestanding layer mode triboelectric nanogenerator. (**b**) The output voltage simulation results when the freestanding layer moves to the right.

As shown in Figure 1a, the left side of the freestanding layer overlaps with the left side of electrode 1 first; the distance that the freestanding layer moves to the right is *x*(*t*) (*x*(*t*) ≥ 0), and the distance between the electrodes is *g*, the width of the electrode is *m*, and the distance from the electrode to the lower surface of the freestanding layer is *h*.

On the premise of  h=0, it was assumed that only a small part of the dielectric surface on the bottom surface contained friction charge *dk*. The charge density is −*σ*, and the total charge on left electrode 1 and right electrode 2 is σmdk. The charge ($dQ_{1}\;\mathrm{and}\;dQ_{2}$) of electrode 1 and electrode 2 in the case of short circuit can be expressed by the following equation, where $C_{i}\left(k\right)$ represents the capacitance between the small interface and electrode i [38]:

(1) $$dQ_{1}=\frac{\sigma m}{1+\frac{C_{2}\left(k\right)}{C_{1}\left(k\right)}}dk$$

(2) $$dQ_{2}=\frac{\sigma m}{1+\frac{C_{1}\left(k\right)}{C_{2}\left(k\right)}}dk$$

According to the principle of the superposition of the electrostatic field, considering that all the charge on the dielectric surface is the superposition of each small electrostatic area, the total charge of electrode 1 and electrode 2 can be expressed in the following form:

(3) $$Q_{1}=\sigma m\int_{0}^{l}\frac{dk}{1+\frac{C_{2}\left(k\right)}{C_{1}\left(k\right)}}$$

(4) $$Q_{2}=\sigma m\int_{0}^{l}\frac{dk}{1+\frac{C_{1}\left(k\right)}{C_{2}\left(k\right)}}$$

So, $\;Q_{sc,final}\;$ can be expressed as:

(5) $$Q_{sc,final}=\int_{0}^{l}\frac{\sigma m}{1+{\left(\frac{C_{2}\left(k\right)}{C_{1}\left(k\right)}\right)}_{x=g+l}}dk-\int_{0}^{l}\frac{\sigma m}{1+{\left(\frac{C_{2}\left(k\right)}{C_{1}\left(k\right)}\right)}_{x=0}}dk$$

When *x* = 0, the distance between the charged dielectric surface and electrode 1 is much smaller than that between electrode 2. According to the capacitance ratio being the inverse of the distance, for any *k* value, the ratio of C2kC1k is close to 0. So, the total charge representation of electrode 1 and electrode 2, $Q_{1}$, is approximately *σml*, and $Q_{2}$ is approximately 0. On the contrary, the distance between the dielectric surface and electrode 2 is much smaller than the distance from electrode 1. Therefore, no matter what value *k* takes, C2kC1k tends to infinity, $Q_{1}\;$ approximates to 0, $Q_{2}$ approximates to *σml*, and the charge transfer efficiency reaches 100%. So, $Q_{sc,final}$ can eventually reach σml, and the change between capacitance ratio and *x* is what drives the flow of electrons between the two electrodes.

Therefore, the relation between maximum transfer charge $Q_{sc,max}$, maximum open circuit voltage $V_{oc,max}$ and capacitance *C_0_* formed between electrodes is as follows:

(6) $$Q_{sc,max}=\sigma ml=C_{0}V_{oc,max}$$

As shown in Figure 1b, the finite element simulation software COMSOL Multiphysics was used to simulate and analyze the freestanding layer mode triboelectric nanogenerator (F-TENG), and the electric potential distribution of the TENG in the working process was verified through simulation. The COMSOL simulation animation of SF-TENG can be observed in Video S1. When the freestanding layer slides from left to right to completely cover the two metal electrodes, the largest positive potential difference and negative potential difference between the two electrodes are obtained, respectively. When the freestanding layer has not touched the electrode and is completely separated from the electrode, the potential difference between the two electrodes is almost zero. When the freestanding layer slides over any electrode, all the charges on the electrode are shielded, so the maximum potential difference can be obtained between the two electrodes. When the freestanding layer is not in contact with the electrodes, the charges on the surfaces of the two electrodes are the same, so the potential difference between the two electrodes is almost zero. This simulation result is consistent with the theoretical analysis above.

### 2.2. Structure and Basic Principles

**Figure 2:**
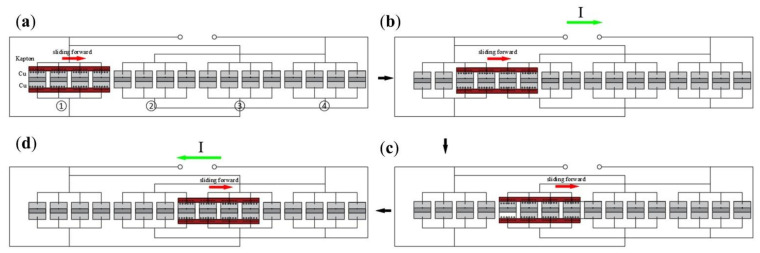
Schematic diagram of the double-layer staggered chain teeth TENG. (**a**) The freestanding layer coincides with electrode ①. (**b**) The freestanding layer moves to the right and contacts the electrode ②. (**c**) The freestanding layer coincides with the electrode ②. (**d**) The freestanding layer moves to the right and contacts the electrode ③.

The structure of one side of the double-layer staggered chain teeth TENG is shown in Figure 2. The size of the double-layer staggered chain teeth TENG chain teeth are shown in Figure S1. The device was mainly composed of two parts: the freestanding layer and staggered chain teeth. The freestanding layer was divided into upper and lower parts, and the surface was composed of a layer of sponge about 1 mm thick, and a layer of Kapton film was attached to the surface of the sponge as the freestanding layer material. The chain teeth shape was cut out with an acrylic board, a copper film was pasted on one surface of the chain-teeth-shaped acrylic board, and the wires were led out. At the beginning, there were 4 chain teeth on one side covered by the freestanding layer, so 4 chain teeth covered by the freestanding layer were used as an electrode.

The working principle of the double-layer staggered TENG can be summarized as the interaction of contact charge and in-plane sliding induction charge. At the beginning, the Kapton layer and the cross-electrode group 1 (electrode ① and electrode ③) (chain teeth) are completely overlapped. When the surface contact friction occurs (the friction polarity of Kapton is much different from that of copper), the negative charge accumulates on the surface of Kapton, and the positive charge accumulates on the surface of electrode ①, and the positive and negative electric charge are equal. The state in Figure 2a belongs to electrostatic equilibrium state, and there is no charge transfer in the circuit at this time. As shown in Figure 2a,b, when the Kapton layer moves to the left from the position coincident with the right electrode group 1 to the electrode group 2 (electrode ② and electrode ④) (chain teeth), the potential of electrode ① is higher than that of electrode ②, and the instantaneous electron moves from electrode ② to electrode ①, resulting in an instantaneous current from electrode ① to electrode ②. When the Kapton layer moves to the position in Figure 2b, the potential difference between electrode ① and electrode ② is zero. Figure S2 shows the overlap of the Kapton layer and the cross-electrode groups 1 and 2. When the Kapton layer moves from Figure 2b to Figure 2c, the potential on electrode ② is higher than that on electrode ①. When moving to the position in Figure 2c, all positive charges are transferred to the electrode group 2, the absolute value of the potential difference between electrode ① and electrode ② reaches the maximum, and the circuit forms another electrostatic equilibrium state. As shown in Figure 2c,d, when the Kapton layer continues to move horizontally to the right, an instantaneous current opposite to the direction of the previous stage is generated. When the Kapton layer moves to coincide with the electrode ③, the current direction changes twice. In this cycle, electrode ① and electrode ② work first, and then electrode ② and electrode ③ work. The double-layer staggered chain teeth TENG was composed of several such parts. The freestanding layer was set to move from left to right, then from right to left, reciprocating the movement once as a movement cycle of the freestanding layer. The period mentioned later was the freestanding layer motion period.

## 3. Results and Discussion

**Figure 3:**
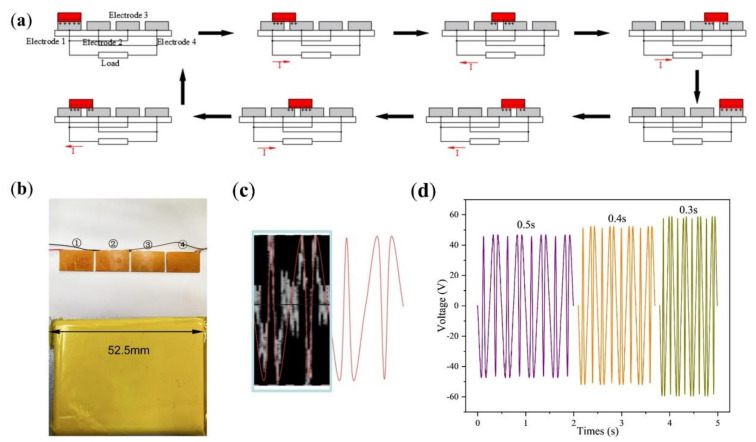
(**a**) Schematic diagram of the staggered electrode TENG. (**b**) Schematic diagram of the electrode connection of the interlaced electrode TENG. (**c**) Voltage variation curve of the freestanding layer cycle of motion for one cycle. (**d**) When the freestanding layer cycle of motion is 0.3 s, 0.4 s and 0.5 s, the output voltage change curve.

According to the design, the chain teeth is divided into front and back surfaces. The surface area of the chain teeth is about 210 mm^2^, and the surface area of eight chain teeth is 1680 mm^2^. Using 32 chain teeth as the experimental object, when the freestanding layer is in motion, the covered surface area of the chain teeth is 1680 mm^2^ (32 mm × 52.5 mm), and the covered area was used as an electrode of TENG. As shown in Figure 3a below, the 32 teeth are divided into 4 similar electrodes, and the double-layer staggered chain teeth TENG was simplified to the staggered electrode TENG. Figure 3b is a physical map of the electrode and the freestanding layer. The distance between electrodes is 4 mm, after beautifying the oscilloscope picture as shown in Figure S3. As shown in Figure 3c, the freestanding layer moves for a cycle of time. The change in the direction of the current corresponds to the situation in Figure 3a, and the extreme point corresponds to when the freestanding layer and the electrode overlap each other. The probe of the oscilloscope was used to connect the electrodes of the staggered electrode TENG; where the electrodes on ① and ③ are connected together, the electrodes on ② and ④ are connected together. The freestanding layer slides back and forth on the electrodes from ① to ④, and other conditions remain unchanged. The time of the freestanding layer sliding back and forth on the electrode was changed. The cycle of the freestanding layer movement was set to 0.3 s, 0.4 s and 0.5 s. The voltage change was observed; as shown in Figure 3d, it can be seen that the output voltage of the staggered electrode TENG decreases with the increase in the freestanding layer movement cycle. When the cycle of the freestanding layer moving on the electrode is 0.3 s, the output voltage amplitude is about 57.5 V.

**Figure 4:**
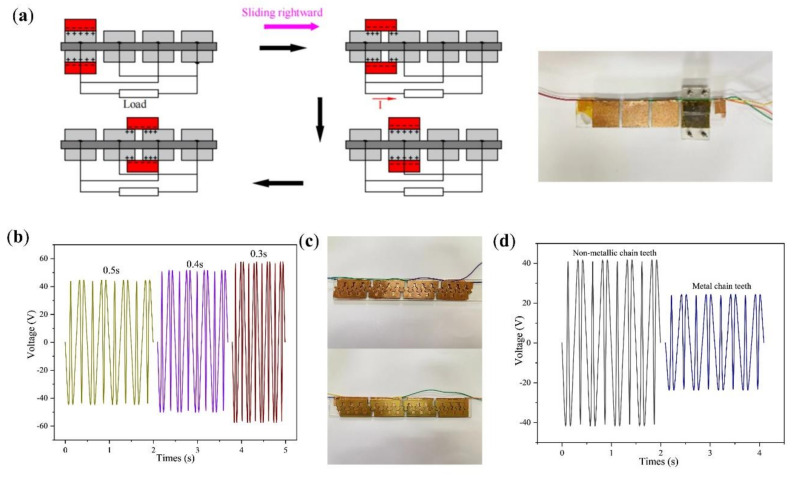
(**a**) Schematic diagram and physical image of the double-layer staggered electrode TENG. (**b**) The voltage change curve of the double-layer staggered electrode TENG when the freestanding layer movement cycle is 0.3 s, 0.4 and 0.5 s. (**c**) Actual picture of the staggered chain teeth TENG with metal teeth and acrylic teeth with the copper film. (**d**) The voltage change curve of the staggered chain teeth TENG with metal teeth and acrylic teeth with the copper film, when the freestanding layer cycle of motion is 0.5 s.

As shown in Figure 4a, the double-layer staggered chain teeth TENG can be simplified again into the double-layer staggered electrode TENG, and the surface area of one electrode is 840 mm^2^ (32 mm × 26.25 mm). Keeping the electrode spacing unchanged and changing the movement cycle of the freestanding layer, the shorter movement cycle of the freestanding layer, the higher the output voltage generated. Comparing Figure 4b with Figure 3d under the same movement cycle, the output voltage generated by the double-layer staggered electrode TENG is slightly smaller. This is because under the same electrode surface area, the number of double-layer connection wires is twice that of the staggered electrode type. When the wires on the same electrode group are connected together, the resistance of the double-layer staggered electrode TENG is slightly greater than that of the staggered electrode TENG. The cycle of the freestanding layer moving on the electrode is 0.3 s, and the output voltage amplitude is about 56.4 V.

According to the above experiments, it can be concluded that the double-layer staggered electrode TENG can work normally. As shown in Figure 4c, the staggered electrode is changed into staggered chain teeth, the spacing between chain teeth is 4 mm, and its output voltage is measured. The chain teeth are divided into metal chain teeth and non-metal chain teeth, and the following experiment is designed, taking a 2 mm thick copper sheet as the chain teeth and comparing it with the copper film pasted on the acrylic as the chain teeth. As shown in Figure 4d, the cycle of the freestanding layer moving back and forth on the electrode is 0.5 s, the output voltage with the metal chain teeth as the output voltage amplitude is about 42.5 V, and the output voltage with the non-metal chain teeth as the output voltage amplitude is about 23.8 V. Since every four non-metallic teeth are connected together, the contact area between the freestanding layer and the electrode (1100 mm^2^) is smaller than the contact area of the staggered electrode TENG (32 mm × 52.5 mm). Under the same cycle, the output voltage of the TENG with four teeth connected together is less than that of the staggered electrode TENG. When using copper sheets as chain teeth, it can be found that, under the same cycle, the effective output voltage value is smaller than the case of the copper film on acrylic. Therefore, a copper film was used as the electrode.

**Figure 5:**
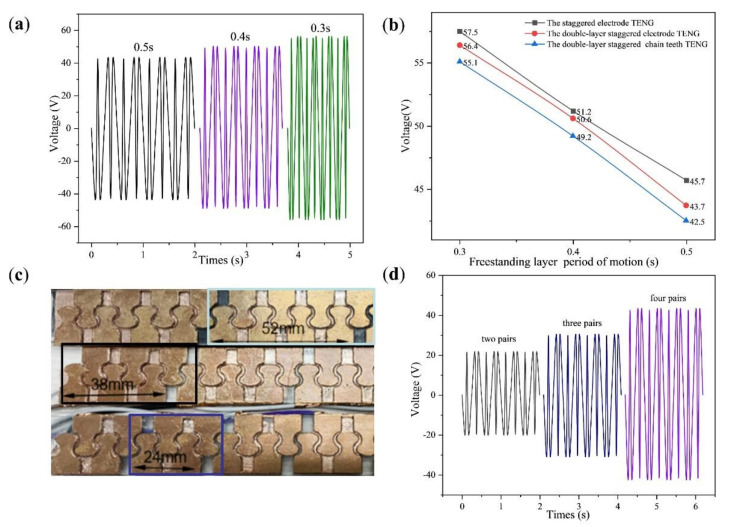
(**a**) The output voltage changes of the double-layer staggered chain teeth TENG under different cycles. (**b**) Comparison between three kinds of TENG output voltage. (**c**) The staggered chain teeth TENG, when the number of chain teeth pairs are 2 pairs, 3 pairs and 4 pairs, respectively. (**d**) The output voltage of the freestanding layer cycle of motion is 0.5 s, when the number of chain teeth pairs are 2, 3 and 4.

Under different cycles, the output voltage change of the double-layer staggered chain teeth TENG is shown in Figure 5a. The staggered electrode TENG, the double-layer staggered electrode TENG and the double-layer staggered chain teeth TENG, under the same conditions, are compared regarding output voltage, as illustrated in Figure 5b. The double-layer staggered chain teeth TENG is smaller than the double-layer staggered electrode TENG, which is smaller than the staggered electrode TENG. Under the same conditions and frequency, the output effect of the staggered electrode TENG and the double-layer staggered chain teeth TENG is not much different. As shown in Figure 5c, the chain teeth of the staggered chain teeth TENG is set as two pairs of staggered chain teeth, three pairs of staggered chain teeth and four pairs of staggered chain teeth as one electrode. Since the range of the freestanding layer initially covers the entire electrode ① and a small part electrode ②, under the same cycle, the output voltage of the double-layer staggered electrode TENG is smaller than the double-layer staggered chain teeth TENG. As shown in Figure 5d, under the same conditions, the measured output voltage increases sequentially.

**Figure 6:**
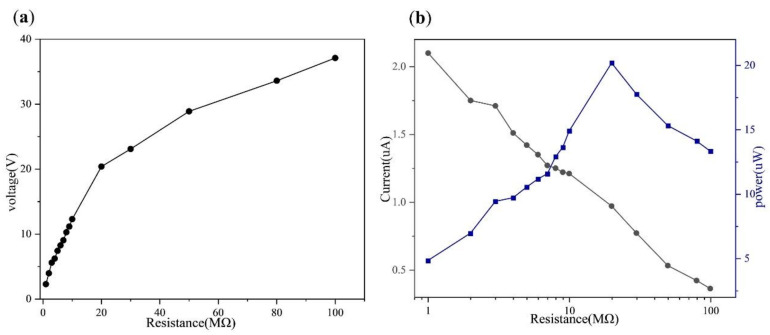
The output performance test of the double-layer staggered chain teeth TENG under different load resistance (**a**) the output voltage when the motion period of the freestanding layer is 0.5 s. (**b**) The output current and power.

According to the above experiment, the copper film is used as the electrode material, and the electrodes are arranged as shown in Figure 6a to form the double-layer chain teeth shape, which is divided into eight electrodes in total. When other conditions remain unchanged, the chain teeth distance and electrode distance are 4 mm, and the reciprocating cycle of the freestanding layer is 0.5 s. The output voltage, current and power of the double-layer staggered chain teeth TENG under different loads resistance are obtained by connecting different external load resistance (1 MΩ~100 MΩ) in series. As shown in Figure 6a, the output voltage is positively correlated with the load resistance. It can be seen from Figure 6b that the output current is negatively correlated with the load resistance. According to the calculation formula between power and load resistance:

(7) $$P=UI=\frac{U^{2}}{R}$$

As shown in Figure 6b, the relationship between output power and external load resistance can be obtained. According to the figure, as the external load resistance increases, the output power first increases and then decreases, and when the external load resistance is 20 MΩ, the output power reaches the maximum value of 20.18 µW. After one month, the double-layer staggered chain teeth TENG continues to be used, and the output voltage of TENG is stable. After the double-layer staggered chain teeth TENG works continuously for 0.5 h, the output voltage is relatively stable, which indicates that the double-layer staggered chain teeth TENG has good stability, as shown in Figure S4.

**Figure 7:**
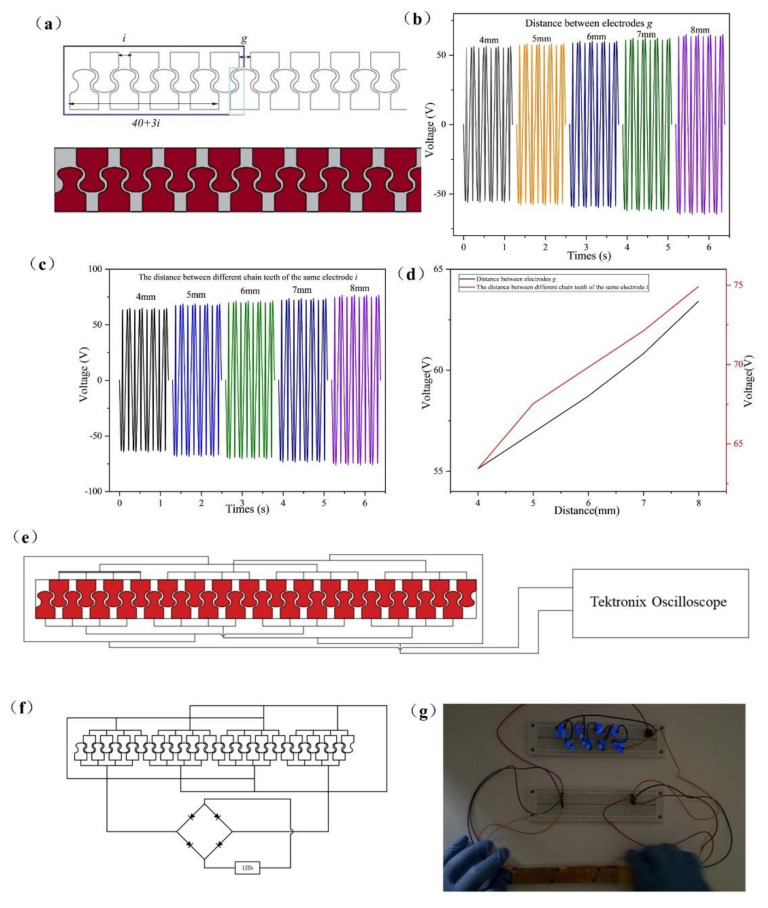
(**a**) The distance *g* between the different electrodes of the double-layer staggered chain teeth TENG, and the distance between the different chain teeth of the same electrode *i*. (**b**) The relationship between the distance *g* between electrodes and the output voltage. (**c**) On the same electrode, the relationship between the distance *i* between different chain teeth and the output voltage. (**d**) The voltage output diagram in two cases. (**e**) The schematic diagram of circuit connection for the double-layer staggered chain teeth TENG. (**f**) The schematic diagram of power management circuit for driving commercial LEDs. (**g**) Simultaneous illumination of 30 LEDs.

Under the same conditions and when the motion period of the freestanding layer is 0.3 s, the electrode spacing g is changed, as shown in Figure 7a, the freestanding layer is slid back and forth, and the output voltage under different electrode distances is obtained, as shown in Figure 7b. When the distance between chain teeth on each electrode is 4 mm, the output voltage increases slightly with the increase in electrode spacing *g* (4~8 mm). The freestanding layer slides back and forth once, and the transferred charge (*Q*) remains unchanged; the short-circuit charge density ($j_{sc}$) and the transferred charge density ($\sigma_{sc}$) are:

(8) $$j_{sc}=\frac{d\sigma_{sc}}{dt}$$

The short-circuit charge density ($j_{sc}$) depends on the length of time spent in a reciprocating motion. When the distance g between adjacent electrodes increases, the movement time of the freestanding layer will increase, which is not conducive to the increase in $j_{sc}$, so the decrease in electrode spacing is conducive to the increase in $j_{sc}$. Reducing the electrode spacing *g* is conducive to the design of high output devices, so 4 mm is the best electrode distance. Assuming that the distance between each electrode is within 5 mm and the distance between two adjacent chain teeth in the same electrode is 8 mm, the adjacent electrodes will interfere, as shown in Figure S5. Therefore, the distance between electrodes changed to 8 mm for the experiment. When the distance between electrodes is 8 mm, the chain teeth distance on each electrode is changed to *i* (4~8 mm), the freestanding layer is slid back and forth, and the experimental data is analyzed. As shown in Figure 7c, under the same frequency, the output voltage increases slightly with the increase in chain teeth distance on each electrode. According to the compactness of the design and the distance between electrodes, the best distance between different chain teeth of the same electrode is 4 mm. Figure 7d shows the comparison curve of the two distances to the output voltage. Figure 7e shows the connection diagram between the double-layer staggered chain teeth TENG and Tektronix Oscilloscope. As shown in Figure 7f, each chain teeth in the double-layer staggered chain teeth TENG can be regarded as zipper teeth, so the double-layer staggered chain teeth TENG can be used as a zipper TENG. On the double-layer staggered chain teeth TENG, the effect of the sliding zipper is simulated, and the output voltage is measured under different cycles. As shown in Figure 7g, connecting the double-layer staggered chain teeth TENG and small lamp beads in series can light up 30 LEDs or more. Figure S6 shows the working diagram of the double-layer staggered chain teeth TENG. As shown in Videos S2 and S3, use the double-layer staggered chain teeth TENG to light up the video of the LED light.

## 4. Conclusions

When there is contact between the freestanding layer and the electrode, the force and the environmental factors remain unchanged, the output performance of the staggered electrode TENG is the best, and the output voltage of the double-layer staggered chain teeth TENG is slightly worse. As the movement speed of the freestanding layer in the staggered electrode TENG, the double electrode TENG and the double-layer staggered chain teeth TENG gradually increases, the output voltage gradually decreases. Two types of chain teeth are designed: metal copper chain teeth and copper film on an acrylic plate. Under the same conditions, the effect of metal chain teeth as electrode output voltage chain teeth is not as good as the copper film as electrode. Staggered chain teeth should choose copper films. When two pairs (four), three pairs (six) and four pairs (eight) of chain teeth are used as an electrode, under the same movement cycle, the output voltage of the double-layer staggered chain teeth TENG increases with the increase in the chain teeth. Therefore, we selected eight chain teeth as TENG electrodes. When the external load resistance is 20 MΩ, the maximum output power reaches 20.18 µW. Under the condition that the contact area of the freestanding layer and the electrode, force, sliding speed and environmental factors remain unchanged, the spacing between the two chain teeth on the same electrode changes (4~8 mm) and the distance between adjacent electrodes changes (4~8 mm) affect the output of the double-layer staggered chain teeth TENG, respectively. The experiment shows that the best electrode spacing and the best chain teeth distance are 4 mm. We also described the optimal chain teeth structure. The structure of the zipper is in the form of chain teeth, which makes it possible to combine the TENG with the zipper.

## Data Availability

Some or all data, models, or code that support the findings of this study are available from the corresponding author upon reasonable request.

## Supplementary Materials

The following supporting information can be downloaded at: https://www.mdpi.com/article/10.3390/mi13030421/s1, Figure S1: Chain teeth size of double-layer staggered chain teeth TENG. Figure S2: The overlapping of the Kapton layer and the cross-electrode groups 1 and 2; Figure S3: Oscilloscope and mapping image. Figure S4: The reliability test of the double-layer staggered chain teeth TENG. Figure S5: If the distance between each electrode is within 5 mm and the distance between adjacent chain teeth in the same electrode is 8 mm, the adjacent electrodes will interfere with each other. Figure S6: Schematic diagram of the work of the double-layer staggered chain teeth TENG. Video S1: The COMSOL simulation animation of SF-TENG. Video S2: When the light was bright, the double-layer staggered chain teeth TENG was used to light up the video of LED light. Video S3: When the light was relatively dark, the double-layer staggered chain teeth TENG was used to light up the video of the LED light.
