# Supplementary material for: Isometric Double-Layer Staggered Chain Teeth Triboelectric Nanogenerator
Source: Micromachines (Basel). 2022 Mar 8;13(3):421. doi: 10.3390/mi13030421 (PMC8954793; doi:10.3390/mi13030421)
Supplement: Supplementary file 1 [file micromachines-13-00421-s001.zip › micromachines-1617013-for final-supplementary.pdf]

micromachines

Supporting Information

Isometric Double-Layer Staggered Chain Teeth Triboelectric Nanogenerator

Shuai Ding, Hua Zhai *, Yaomin Shao and Rui Lei

Supplementary Figures:

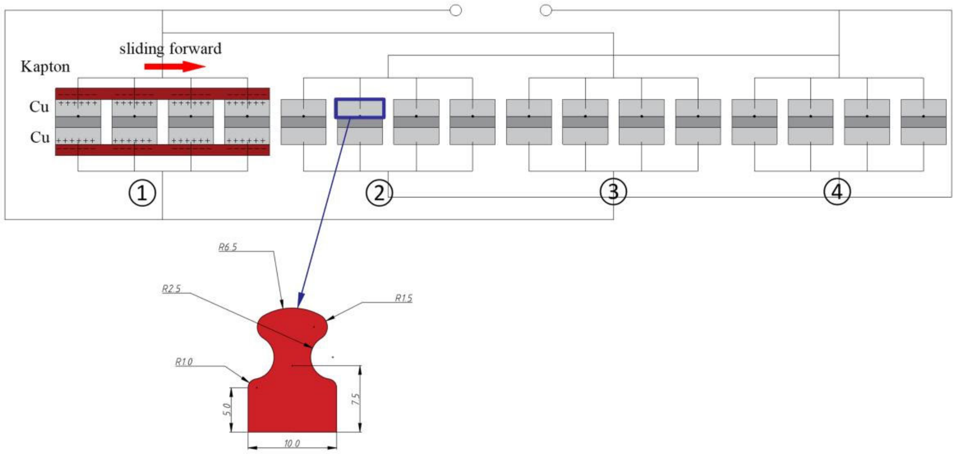

Figure S1. Chain teeth size of the double-layer staggered chain teeth TENG.

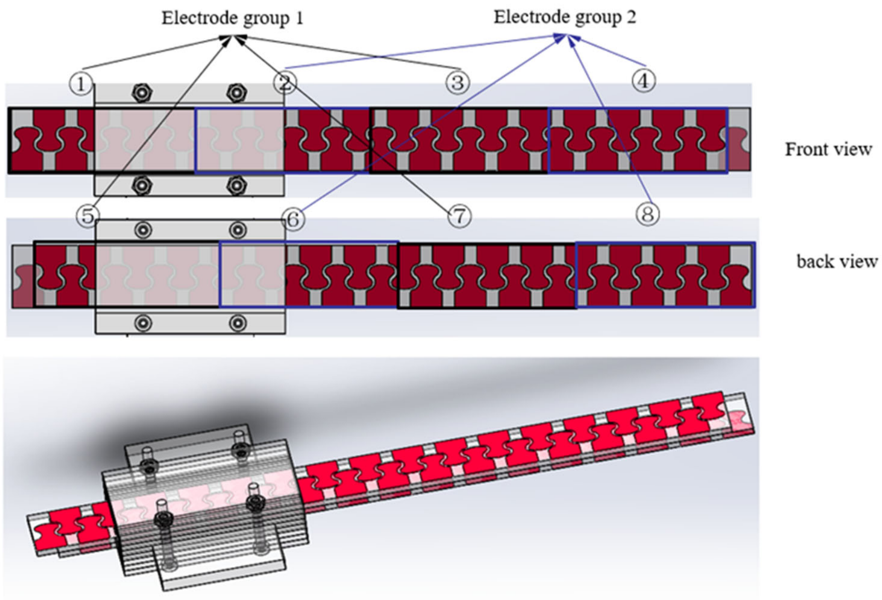

Figure S2. The overlapping of the Kapton layer and the cross-electrode groups 1 and 2.

Citation: Ding, S.; Zhai, H.; Shao, Y.; Lei, R. Isometric Double-Layer Staggered Chain Teeth Triboelectric Nanogenerator. *Micromachines* **2022**, *13*, 421. https://doi.org/10.3390/mi13030421

Academic Editors: Hao-Yang Mi, Xin Jing and Bao Yang

Received: 14 February 2022
Accepted: 07 March 2022
Published: date

Publisher's Note: MDPI stays neutral with regard to jurisdictional claims in published maps and institutional affiliations.

Copyright: © 2022 by the authors. Submitted for possible open access publication under the terms and conditions of the Creative Commons Attribution (CC BY) license (https://creativecommons.org/licenses/by/4.0/).

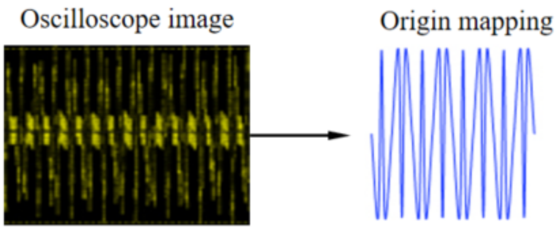

Figure S3. Oscilloscope and mapping image.

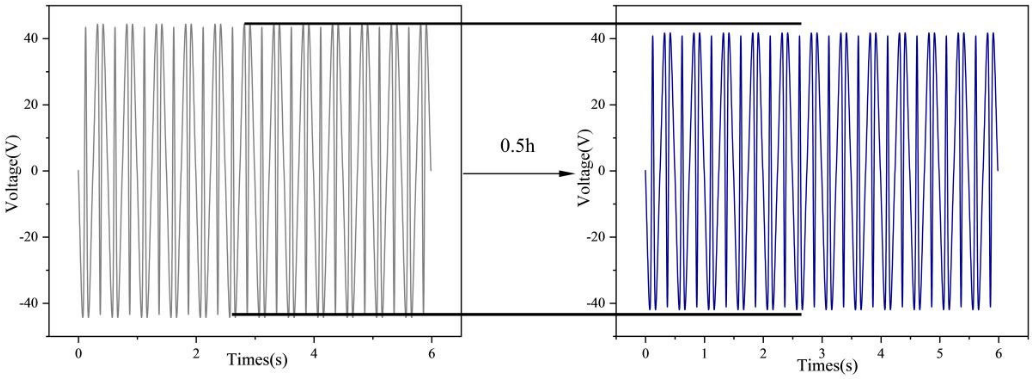

Figure S4. The reliability test of the double-layer staggered chain teeth TENG.

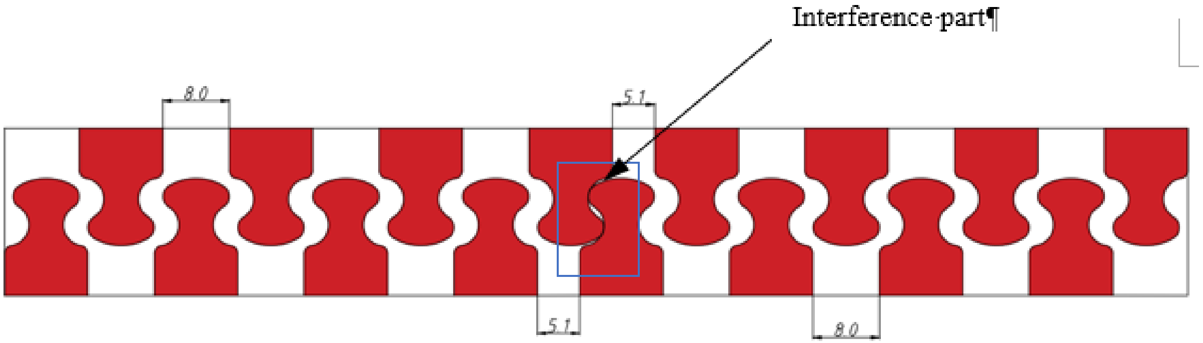

Figure S5. If the distance between each electrode is within 5mm and the distance between adjacent chain teeth in the same electrode is 8mm, the adjacent electrodes will interfere with each other.

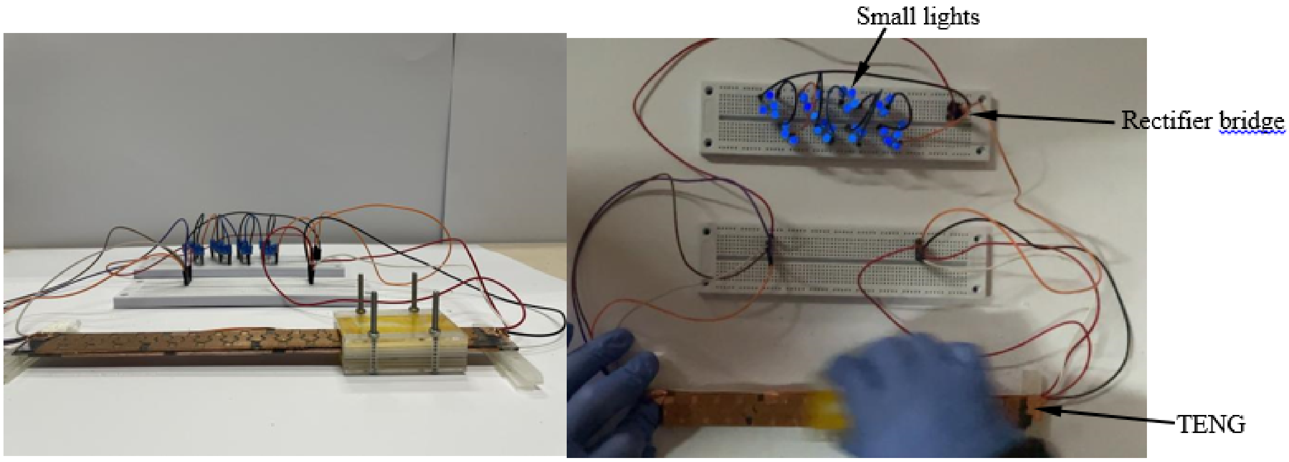

Figure S6. Schematic diagram of the work of the double-layer staggered chain teeth TENG

Supplementary Videos:

Video S1: The COMSOL simulation animation of SF-TENG.

Video S2: When the light is bright, use the double-layer staggered chain teeth TENG to light up the video of LEDs light.

Video S3: When the light is relatively dark, use the double-layer staggered chain teeth TENG to light up the video of the LEDs light.
